# Smear positive pulmonary tuberculosis among diabetic patients at the Dessie referral hospital, Northeast Ethiopia

**DOI:** 10.1186/2049-9957-2-6

**Published:** 2013-03-27

**Authors:** Hiwot Amare, Aschalew Gelaw, Belay Anagaw, Baye Gelaw

**Affiliations:** 1 Bahir Dar Regional Health Research Laboratory Center, Bahir Dar, Ethiopia; 2 Department of Medical Microbiology, School of Biomedical and Laboratory Sciences, College of Medicine and Health Sciences, University of Gondar, Gondar, Ethiopia

**Keywords:** Dessie, Diabetic patients, Ethiopia, Pulmonary tuberculosis (PTB)

## Abstract

**Background:**

Tuberculosis (TB) is an infectious disease which is still a major cause of morbidity and mortality throughout the world. People with diabetes mellitus (DM) have a three times higher risk of developing active TB than people without diabetes. However, there is not enough credible information on the burden of pulmonary tuberculosis (PTB) among DM patients in Ethiopia, in general, and in the city of Dessie, in particular. Therefore, this study aims to determine the prevalence and associated risk factors of smear positive PTB among diabetic patients at a referral hospital in Dessie.

**Methods:**

A cross-sectional study was conducted from February 2012 to April 2012. Patient demographic characteristics were collected using a pre-tested standard questionnaire format. Spot-morning-spot sputum specimens were collected from the study participants and examined for acid-fast bacilli using direct microscopy by the Ziehl-Neelsen staining technique. Data was entered and analyzed using the SPSS version 16 statistical software and p-value <0.05 was considered as statistically significant.

**Results:**

Out of 225 TB suspected diabetic patients, 52% were males and 48% were females. Their ages ranged from 12 to 82 years, with a mean age of 47.2 years. Urban residence (AOR: 5.5; 95% CI: 1.07–28.20), history of TB (AOR: 13.4; 95% CI: 2.74–65.73), contact with TB patients in the family (AOR: 9.4; 95% CI: 1.822–48.50), and long duration of DM (AOR: 8.89; 95% CI: 1.88–58.12) were independently associated with the development of active TB in people living with DM.

**Conclusions:**

The prevalence of smear positive PTB was 6.2% in TB suspected diabetic patients, which is higher compared with the general population (0.39%). Patients with a previous history of contact with TB patients, as well as those who had prolonged diabetes, were more prone to have PTB. Therefore, screening of diabetic patients for PTB infection during follow-up is necessary.

## Multilingual abstracts

Please see Additional file 1 for translations of the abstract into the six official working languages of the United Nations.

## Background

Tuberculosis (TB) is an infectious disease caused by various strains of mycobacteria, especially *Mycobacterium tuberculosis* and usually attacks the lung [1]. It remains to be a major cause of morbidity and mortality throughout the world. It is estimated that one-third of the world's population is infected, 8.8 million people develop TB, and 1.45 million people die annually from the disease [2]. In Africa, about 2.8 million incident TB cases and 390 thousands TB deaths occurred in 2009 [3]. Ethiopia ranks seventh among the world’s 22 high-burden TB countries [2]. According to the World Health Organization’s (WHO) Global TB Report 2011, Ethiopia had an estimated incidence rate of 261 cases per 100,000 population and 29 thousands deaths in 2010, with an estimated prevalence rate of 394 cases per 100,000 population [2]. The incidence of TB was reported greatest among people with impaired immunity, HIV infection, or diabetes [4].

Diabetes mellitus (DM) is complex metabolic disorder that is characterized by a high level of blood sugar either because the body does not produce enough insulin or cells do not respond to the insulin [5]. This high level of blood sugar produces the classical symptoms of frequent urination, and increased thirst and hunger. The total number of people with DM worldwide is projected to rise from 285 million in 2010 to 439 million in 2030 [4]. In 2010, 12.1 million people were estimated to be living with diabetes in Africa, and this is projected to increase to 23.9 million by 2030 [6]. The WHO estimated that the number of cases of diabetes in Ethiopia was about 800,000 in 2000, and projected that it would increase to about 1.8 million by the year 2030 [7].

Besides HIV, malnutrition, alcoholism, and smoking, DM has received recent recognition as a risk factor for TB [8]. Epidemiological studies have elucidated an association between DM and the development of TB [9]. According to a study done by Jeon CY et al., people with DM had approximately a three times higher risk of developing TB than people without diabetes, and this has been evidenced by biological support of the causal relationship between diabetes and host immunity to TB [10]. Evidence from an experimental study revealed that diabetes was associated with reduced macrophage functions, such as chemotaxis, phagocytosis, and bactericidal actions, and also impairs the function and proliferation of T-helper 1 cells and their production of cytokines [11].

On the other hand, a study done in Brazzaville, Congo showed that TB patients with diabetes were more likely to have an increased rate of treatment failures, deaths, defaults and relapses, delayed smear, and culture conversion [12]. It was also found that TB treatment reduced the effectiveness and concentration of diabetes medications, which makes it difficult to control diabetes [13]. However, the prevalence of PTB among diabetic patients was not assessed in the study area. Therefore, this study was conducted to determine the magnitude of smear positive PTB and its associated risk factors among TB suspected diabetic patients at the Dessie referral hospital.

## Methods

### Study design and period

A cross-sectional study was employed to assess the prevalence and associated risk factors for the occurrence of PTB among TB suspected diabetic patients from February 2012 to April 2012.

### Study area and participants

The study participants were recruited from the Dessie referral hospital. Dessie is located in the northeast part of Ethiopia in the South Wollo administrative zone of the Amhara Region, which is 401 km away from the capital city, Addis Ababa. This referral hospital provides services to the population in the surrounding area of the town and the adjacent regions. In this study, 225 TB suspected diabetic patients who visited the Dessie referral hospital during the study period were included.

### Sample size and sampling technique

The sample size was determined using a single population proportion formula, n = z^2^p (1-p)/ w^2^, where n = sample size, Z = standardized normal distribution value at the 95% CI, which is 1.96; P = the proportion of TB sufferers among diabetics from a previous study conducted at Addis Ababa, 5.8% [14], and w = the margin of error, taken as 3%. Since the average total number of diabetic patients who visited the Dessie referral hospital for follow-ups was approximately 1,700 (i.e. < 10,000), the required maximum sample of 205 was obtained from the above estimate by making some adjustments. The final sample size was determined to be 236 after calculating that nf = n/1 + (n/N) and by taking into account a 15% non-response rate.

### Sampling technique

All pulmonary tuberculosis (PTB) suspected diabetic patients who visited the Dessie referral hospital during the study period were included until the required sample size (236) was obtained.

### Data collection tools

#### Administration of questionnaire

Information concerning patients' socio-demographics and associated factors was collected by trained nurses using a pre-tested standard questionnaire during the study period.

#### Specimen collection and laboratory investigation

Diagnosis of smear positive PTB among TB suspected diabetic patients were done based on the national TB diagnosis guidelines. All laboratory investigations were done adhering to accepted standard procedures. Three consecutive sputum samples (spot-morning-spot) were collected, smeared, and stained with the Ziehl-Neelsen staining method. The stained smears were then examined under the oil immersion objective to look for acid-fast bacilli in a light microscope.

As a quality control method, all new lots of reagents were tested with known positive and negative control slides, and then all the positive and 10% of the negative slides were re-read by experienced laboratory technologists who were bind for the smear for confirmation. Pulmonary TB is diagnosed if at least two smear results are positive for AFB or one sputum specimen is positive with additional x-ray abnormality [15]. Fasting blood glucose was measured by a photometer and a glucometer based on the procedure supplied by the manufacturer.

#### Data processing and analysis

The data was entered and analyzed using the statistical package for social sciences (SPSS) version 16 statistical software. Odds ratios (OR) and their 95% confidence intervals (CI) were estimated using bivariate and multivariate logistic regression analysis to identify possible explanatory variables on occurrence of PTB. The result at p-value <0.05 was considered as statistically significant.

### Ethical considerations

Before starting the study, ethical clearance was obtained from the ethical review committee of the School of Biomedical and Laboratory Sciences, University of Gondar. Informed consent was also obtained from the Dessie referral hospital and the study participants. The confidentiality of the information collected was maintained by using code numbers for participants. In addition, the clinical specimens collected during the study period were used for the stated objectives. For those participants who were positive for PTB, appropriate treatment was prescribed by physicians and health education was given to prevent spread of the infection to others.

## Results

### Socio-demographic characteristics of tuberculosis suspected diabetic patients

A total of 236 TB suspected known diabetic patients were expected to be enrolled in this study. However, 11 (4.7%) respondents who were unable to bring sufficient sputum for investigation were excluded. As a result, this study had a response rate of 95.3%. Of the 225 study participants, 116 (51.56%) were males and the mean age was 47.2 years (ranging from 12–82 years). The majority (147) of the participants (65.4%) were above the age of 40. More than half the respondents (144) were married (64%) and 139 were urban dwellers (61.8%). In terms of literacy, 141 of the respondents (62.7%) were illiterate and just completed elementary school; and the rest either completed high school or higher education. Regarding occupational status, 77 respondents (34.22%) were employed (government or private), 61 were merchants (27.1%), 61 were farmers (27.1%), and the remaining 26 (11.6%) were laborers. The monthly income of 67 respondents (29.8%) was less than US 23 dollars, followed by 56 respondents (24.9%) who earn US 38–67 dollars (see Table 1).

**Table 1:** Socio-demographic characteristics of tuberculosis suspected diabetic patients at the Dessie referral hospital, from February to April 2012

***Characteristics***	**PTB infection**		**Frequency (N = 225)**
	**Negative n (%)**	**Positive n (%)**	**n (%)**
**Sex**			
Male	105 (90.5)	11 (9.5)	**116 (51.5)**
Female	106 (97.2)	3 (2.8)	109 (48.5)
**Age (years)**			
≤20	18 (97.7)	1 (2.3)	19 (8.4)
21–30	19 (95.0)	1 (5.0)	20 (8.9)
31–40	36 (92.3)	3 (7.7)	39 (17.3)
41–50	55 (91.7)	5 (8.3)	60 (26.7)
>50	83 (95.4)	4 (4.6)	**87 (38.7)**
**Religion**			
Christian	106 (97.2)	3 (2.8)	109 (48.4)
Muslim	105 (90.5)	11 (9.5)	**116 (51.6)**
**Education level**			
Illiterate	74 (94.8)	4 (5.2)	**78 (34.7)**
Elementary school	60 (95.2)	3 (4.8)	63 (28)
Secondary school	32 (89)	4 (11)	36 (16)
Higher education	45 (93.7)	3 (6.3)	48 (21.3)
**Marital status**			
Single	41 (97.6)	1 (2.4)	42 (18.7)
Married	135 (93.7)	9 (6.3)	**144 (64)**
Divorced	17 (89.4)	2 (10.6)	19 (8.4)
Widowed	18 (90)	2 (10)	20 (88.9)
**Place of residence**			
Urban	128 (92)	11 (8)	**139 (61.8)**
Rural	83 (96.5)	3 (3.5)	86 (38.2)
**Occupation**			
Employed *	71 (92.2)	6 (7.8)	77 (34.2)
Farmer	59 (96.7)	2 (3.3)	61 (27.1)
Merchant	59 (96.7)	2 (3.3)	61 (27.1)
Laborer	22 (84.6)	4 (15.4)	26 (11.6)
**Average monthly income (US dollars)**			
<23	64 (95.5)	3 (4.5)	**67 (29.8)**
23–38	46 (95.8)	2 (4.2)	48 (21.3)
39–67	52 (92.9)	4 (7.1)	56 (22.7)
>68	49 (90.7)	5 (9.3)	54 (24.3)

* = employed by the government or privately.

Coughing was the most common symptom, with all the respondents reporting this symptom. This was followed by 220 respondents reporting sputum production (97.8%), 104 had loss of appetite (46.2%), 72 had night sweating (32%), and 54 had chest pain (24%). The duration of DM in all patients varied from one year to 30 years, with a mean duration of 5.6 ± 4.4 years. More than half of the respondents (132) had DM for less than five years (58.7%). The body mass index (BMI) of the TB suspected diabetes patients showed that 141 respondents (62.7%) were normal (18.5–24.99 kg/m^2^), 30 (13.3%) were underweight (<18.5 kg/m^2^) and 54 (24%) were overweight (≥25 kg/m^2^) (see Table 2).

**Table 2:** The patterns of pulmonary tuberculosis and types of diabetes, body mass index, and blood glucose levels of respondents at the Dessie referral hospital, from February to April 2012

***Characteristics***	**TB infection among patients**		**Total (%)**
	**Negative (%)**	**Positive (%)**	
**Types of DM**			
Type 1	37 (92.5)	3 (7.5)	40 (17.8)
Type 2	174 (94.1)	**11 (5.9)**	**185 (82.2)**
**Body mass index (kg/m**^**2**^**)**			
<18.5	29 (96.7)	1 (3.33)	30 (13.3)
18.5–24.99	130 (92.2)	**11 (7.8)**	**141 (62.7)**
≥25	52 (96.3)	2 (3.7)	54 (24)
**Blood glucose (mg/dl)**			
≤168	57 (98.3)	1 (1.7)	58 (25.8)
169–220	55 (96.5)	2 (3.5)	57 (25.3)
221–288	51 (94.4)	3 (5.6)	54 (24)
>288	48 (85.7)	**8 (14.3)**	**56 (24.9)**

### Prevalence of pulmonary tuberculosis among diabetic patients

Out of the 225 TB suspected diabetic patients, *Mycobacterium tuberculosis* was detected in 14 of the diabetic patients (11 males and three females) by direct microscopy, and supported by chest x-ray. Therefore, the overall prevalence of smear positive PTB was 6.2% in the study population. In addition, five (35.7%) of these TB-diabetic patients were also infected with HIV.

A relatively higher proportion of PTB was observed among the age group of 40 years and over [64.3% (9/14)], in male respondents [78.6% (11/14)], and in urban dwellers [78.6% (11/14)]. Data on marital status showed that 64.3% (9/14) of PTB positive patients were married, 28.6% (4/14) were divorced or widowed, and the rest were single. Most of the PTB patients were also employed 42.8% (6/14). Most of the TB-diabetes patients had type 2 diabetes 78.6% (11/14) and type 1 diabetes accounted for 21.4% (3/14).

### Risk factors associated with pulmonary tuberculosis

Bivariate logistic regression was used to identify possible explanatory (independent) variables and those variables, which have a p-value of less than 0.20, were taken to multivariate logistic regression. As a result, place of residence (P = 0.041), religion (P = 0.049), past history of TB (P = 0.001), presence of TB patients in the family (P = 0.012), and duration of diabetes (P = 0.008) were significantly associated with the occurrence of PTB (see Table 3). On the other hand, age (P = 0.811), sex (P = 0.103), educational status (P = 0.619), occupational status (P = 0.659), marital status (P = 0.921), monthly income (P = 0.666), blood glucose level (P = 0.267), smoking (P = 0.410), BMI (P = 0.463), and consumption of alcohol (P = 0.466) were not significantly associated with development of PTB.

**Table 3:** Association of socio-demographic characteristics and other risk factors with pulmonary tuberculosis in respondents at the Dessie referral hospital, from February to April 2012

***Characteristics***	**PTB infection**		**COR(95% CI)**	**AOR(95% CI)**	**P-value**
	**−ve(n)**	**+ve(n)**			
**Sex**					
Male	105	11	3.7(1.04–13.65)	3.6(0.77–16.73)	0.103
Female	106	3	1.00	1.00	
**Religion**					
Christian	106	3	1.00	1.00	0.034
Muslim	105	11	3.7(1.04–13.648)	5.03(1.13–22.42)	
**Place of residence**					
Urban	128	11	2.38(0.64–8.778)	**5.5**(1.07–28.20)	0.041
Rural	83	3	1.00	1.00	
**Smoking**					
Yes	5	2	6.87(1.21–39.12)		
No	206	12	1.00		
**Past history of TB**					
Yes	20	6	7.16(2.26–22.72)	**13.4**(2.74–65.73)	0.001
No	191	8	1.00	1.00	
**Presence of TB patients in the family**					
Yes	23	5	4.54(1.40–14.72)	**9.4**(1.82–48.50)	0.007
No	188	9	1.00	1.00	
**Duration of DM**					
≤5 years	129	3	1.00	1.00	0.025
6–10 years	56	5	3.84(0.88–16.62)	4.01(0.48–16.43)	
>10 years	26	6	9.92(2.33–42.24)	**8.89**(1.88–58.12)	

−ve = negative.

+ve = positive.

Urban dwellers were about six times (AOR = 5.5; 95% CI = 1.07–28.20) more likely to develop PTB than rural residents. Respondents who had a previous history of TB were also 13 times (AOR = 13.4; 95% CI = 2.74–65.73; P = 0.001) more likely to develop PTB than those who did not have a previous history of TB. Regarding family history of TB, those who had contact with TB patients were about nine times (AOR = 9.4; 95% CI = 1.82–48.50; P = 0.007) more likely to develop PTB than respondents who didn’t have history of contact with TB patients (see Table 3).

**Figure 1:**
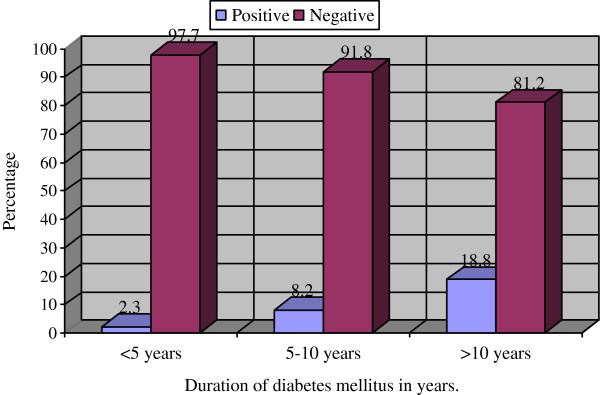
Prevalence of smear positive pulmonary tuberculosis compared with the duration of diabetes mellitus in patients at the Dessie referral hospital, from February to April 2012.

Moreover, the duration of diabetes was independently associated with PTB. The prevalence of PTB increased progressively with the duration of DM (see Figure 1). High prevalence of PTB was observed among the study population who had diabetes for more than ten years (2.7%) followed by those who had diabetes for five to ten years, and less than five years (2.2% and 1.3%, respectively). Diabetic patients that had the disease for more than ten years were nine times (AOR = 8.89; 95% CI = 1.88–58.12; P = 0.025) more likely to develop PTB than those who have lived with DM for less than five years.

Although, the BMIs and blood glucose levels of the respondents were not significantly associated with the development of PTB, relatively high prevalence of PTB was observed among TB suspected diabetic patients who had a normal BMI (7.8%) and a blood glucose level greater than 288 mg/dl (14.3%). The prevalence of PTB among malnourished (<18.5 kg/m^2^) and overweight (≥25 kg/m^2^) diabetes patients was 3.3% and 3.7%, respectively (see Table 2).

## Discussion

Previous studies have identified an important association between diabetes and TB, in that DM is one of the risk factors for the development of active PTB [8,9]. However, in Ethiopia, particularly in Dessie, little is known about prevalence and associated risk factors of PTB in the population of those with diabetes. Hence, the present study tries to provide insights into the prevalence of PTB in diabetic patients, as well as outline some possible risk factors.

In this study, the prevalence of PTB among TB suspected diabetic patients was 6.2%. It is higher than the 2011 WHO estimated prevalence of TB in the general population of 0.39% [3]. This finding is in line with the reports from Tanzania (5.4%) and India (6%) [16-18]. However, the finding of the current prevalence of PTB among diabetics was higher than the prevalence reported in Korea (2.12%) [19]. On the other hand, the burden of PTB in Dessie was lower than the prevalence reported from Pakistani studies that revealed the prevalence of PTB to be from 9.5% to 14% among diabetic patients [20-22]. This variation could be due to the use of an advanced diagnosis technique in Pakistani studies, and the fact that the study participants were admitted patients and thus were more likely to be positive.

A study done in Addis Abeba showed that 4.14% of diabetic patients had PTB, which is lower than the present study finding [7]. The increased prevalence of PTB in our study might be due the involvement of TB suspected diabetic patients as compared to the study in Addis Ababa, which was retrospective and considered all diabetic patients.

The socio-demographic characteristics of PTB suspected diabetic patients and other risk factors for active PTB infection were also investigated. Several studies have shown that socio-economic status is a risk factor for active TB occurring [23-25]. The result of this study showed that the higher the age of the patients, the more likely it is that they will have TB infection if they are also diabetic. The majority of the patients who developed PTB were older than 40 years of age and this was comparable to other age reports from Indian, Korea, and Pakistan [17,20,22]. The possible reason for this may be due to a compromised host’s immunity that increased susceptibility to TB due to aging, and most diabetic patients were in the over 40 age group as well.

The association of sex with active PTB infection was not statistically significant. However, among the 14 PTB patients, 11 (78.6%) were male which indicates a higher proportion of PTB infection among male diabetic patients rather than females (21.4%). This result was consistent with a study in Pakistan (78% versus 22%, respectively) [22].

Urban residence was also associated with the development of PTB. The prevalence of smear positive PTB in this study was higher in urban dwellers (52.7%) than rural residents. This finding was consistent with a study done in India, in which the prevalence of smear-positive TB was reported to be 69.2% [26]. The possible reasons for this could be because of crowded living conditions in urban areas, as well as urban dwellers having lower levels of physical activity and mostly consuming a calorie rich diet, which increases fat accumulation in the body. Physically inactive or sedentary lifestyles lead to excess body fat accumulation which increases insulin resistance and ultimately results in higher blood glucose levels which impairs the immune cells against TB infection [23].

The role of smoking in the development of active TB is well established [27], but smoking was not associated with active TB in this current study and this could probably be attributed to a social desirability bias whereby smokers denied their smoking status.

This study also identified the variable that has the most important influence on the occurrence of PTB. It revealed that patients who had a previous history of TB were significantly associated with developing smear positive PTB than those who did not have a previous history of TB. Moreover, living with a TB patient had been one of the most important predictors of smear positive PTB in this study, which is consistent with previous findings in Pakistan and India [17,20,28]. This might be due to the fact that frequent contact with TB patients in a household could lead to increased transmission of PTB.

The duration of DM has been also associated with the risk of developing smear positive PTB. Those patients who have had DM for more than ten years had a higher proportion of PTB than those who had shorter durations (<5 years) of DM, which is consistent with a study done by Jabbar et al. that showed a high prevalence of PTB among diabetic patients who had DM for more than ten years [21]. Uncontrolled high levels of blood glucose for long periods of time could be another factor associated with the development of TB. In addition, in low-resource settings, early diagnosis and adequate glycemic control of DM is difficult, and this will probably further increase the proportion of diabetic patients developing TB.

In general, the present study and other studies carried out so far show the high burden of PTB among the diabetic populations. Therefore, active screening and treatment of PTB among patients with DM is especially relevant in TB-endemic countries, such as Ethiopia.

This study had some limitations. Direct smear microscope alone may underestimate the prevalence of PTB in the study population. We believe that comparing the prevalence of PTB with non-diabetic patients to that of diabetic patients might disclose important information about the occurrence of PTB. However, due to financial constraints, we were unable to determine the prevalence of PTB among non-diabetic patients. Selection bias may also arise from convenience sampling.

## Conclusion

The overall prevalence of smear positive PBT among TB suspected diabetic patients was high (6.2%) in Dessie. Pulmonary TB was occurring more often among patients with DM (>10 years), and among patients who had high blood glucose levels. Patients with a previous history of TB, history of contact with TB patients in the family, living in urban areas, and prolonged duration of DM were independent risk factors for the occurrence of active PTB. Therefore, we recommend that, at the time of their diagnosis, diabetic patients be screened for PTB and have regular check-ups. Further studies are also recommended to strengthen and explore the problem among diabetic patients in depth with large sample sizes and advanced diagnostic techniques.

## Competing interests

There was no conflict of interest among the authors or with any other parties.

## Authors’ contributions

HA conceived, designed, and proposed the research idea. HA, AG, BA, and BG were all involved in data collection, entry, clearance, analysis, and interpretation of the findings. HA was responsible for drafting the manuscript. All authors were involved in reviewing the manuscript and approving it for publication.

## Supplementary Material

Additional file 1 Multilingual abstracts in the six official working languages of the United Nations. Click here for file
