# Supplementary material for: Smear positive pulmonary tuberculosis among diabetic patients at the Dessie referral hospital, Northeast Ethiopia
Source: Infect Dis Poverty. 2013 Mar 27;2:6. doi: 10.1186/2049-9957-2-6 (PMC3707095; doi:10.1186/2049-9957-2-6)
Supplement: Additional file 1 — Multilingual abstracts in the six official working languages of the United Nations. [file 2049-9957-2-6-S1.pdf]

السل الرئوي الإيجابي اللطاخة بين مرضى السكري في في مستشفى ديسي للإحالة، شمال شرق إثيوبيا

هيووت أمار، أشاليو غيلاو، بيلاي أناغاو، باي غيلاو

الملخص

السل مرض معد من الأمراض التي تشكل أحد أكبر مصادر المراضة والوفيات في جميع أنحاء العالم. وتتضاعف إمكانية إصابة مرضى داء السكري بالسل الفعال ثلاث مرات مقارنة بغير المصابين بالسكري. ولكن لا تتوفر معلومات موثوقة عن تأثير السل الرئوي على المصابين بداء السكري في إثيوبيا بشكل عام وفي ديسي بشكل خاص. لذا تهدف هذه الدراسة إلى تحديد معدل انتشار وعوامل الخطورة المصاحبة لدى إيجابيي لطاخة السل الرئوي بين مرضى السكري في مستشفى الإحالة في ديسي. أجريت دراسة مستعرضة شاملة لكل القطاعات والفئات ابتداء من فبراير 2012 وحتى أبريل 2012. جمعت الخصائص الديمغرافية للمرضى باستخدام نموذج استقصاء قياسي مسبق الاختبار. تم جمع عينات قشع اللقاء الأول- الصباح- موعد المراجعة من المشاركين في الدراسة ثم تم فحصها بحثا عن العصيات المثبتة للحامض بالفحص المجهري المباشر باستخدام تقنية تلوين تسيل-نيلسن. تم إدخال البيانات وتحليلها باستخدام برمجية SPSS 16 مع اعتبار عامل الاحتمالية $p<0.05$ مميز إحصائيا. من بين 225 مريض بالسكري ومشتبه بإصابته بالسل، كان 52% منهم من الذكور و48% من الإناث. وتراوحت أعمارهم بين 12 و 82 سنة وكان معدل العمر الوسطي 47.2 سنة. كان معدل انتشار السل الرئوي الإيجابي اللطاخة 6.2%. السكن في المدن (نسبة الأرجحية المعدلة: 5.5، 95%، مدى الثقة: 1.07 – 28.20)، وجود إصابة سابقة بالسل (نسبة الأرجحية المعدلة: 13.4، 95%، مدى الثقة: 2.74 – 65.73)، الاتصال مع مريض مصاب بالسل في الأسرة (نسبة الأرجحية المعدلة: 9.4، 95%، مدى الثقة: 1.82 – 48.50)، والإصابة بالسكري لوقت طويل (نسبة الأرجحية المعدلة: 8.89، 95%، مدى الثقة: 1.88 – 58.12) ترافقت بشكل مستقل مع تطور السل الفعال لدى الأشخاص المصابين بالسكري. كان معدل انتشار السل الرئوي الإيجابي اللطاخة (6.2%) بين مرضى السكر المشتبه بإصابتهم بالسل، وهي نسبة أعلى إذا ما قورنت بمعدل الحدوث بين إجمالي السكان (0.394%). المرضى الذي كان لديهم سوابق إصابة بالسل أو تواصل مع مصابين بالسل أو إصابة مديدة بالسكري كانوا أكثر عرضة للإصابة بالسل الرئوي. لذا، من الضروري إجراء مسح استقصائي لمرضى السكري للكشف عن الإصابة بعدوى السل أثناء متابعة حالاتهم.

Translated from English version into Arabic by Lina SM, through

TRANSLATORS
WITHOUT BORDERS

埃塞俄比亚东北部 Dessie 市复诊医院中糖尿病患者的涂片阳性肺结核调查

Hiwot Amare, Aschalew Gelaw, Belay Anagaw, Baye Gelaw

摘要

结核病仍是全球发病和死亡的主要传染病之一。糖尿病患者得活动期结核病的风险比非糖尿患者高 3 倍。但在埃塞俄比亚，特别是 Dessie 市，还没有足够可靠的糖尿病患者的肺结核负担的相关资料。因此，于 2012 年 2 月至 4 月进行横向研究，以确定 Dessie 市一所复诊医院中，糖尿病患者的涂片阳性肺结核的发病率和相关风险因子。通过预测试标准调查问卷收集患者的个人信息。采集参与调查患者的斑晨痰标本，经 Ziehl-Neelsen 染色后，于显微镜下观察抗酸杆菌。数据采用 SPSS 16 软件分析，$P<0.05$ 为差异有统计学意义。225 例疑似肺结核的糖尿病患者中，52%为男性，48%为女性。年龄为 12–82 岁，平均年龄为 47.2 岁。城市居民(AOR: 5.5; 95%CI: 1.07–28.20)、结核病史(AOR: 13.4; 95%CI: 2.74–65.73)、与家中结核患者接触(AOR: 9.4; 95%CI: 1.822–48.50)和糖尿病时间长(AOR: 8.89; 95%CI: 1.88–58.12)均各自独立地与糖尿病患者的活动期结核病发展有关联。疑似肺结核的糖尿病患者中，涂片阳性肺结核的发病率为 6.2%，高于普通人群(0.394%)。有接触结核病患者史和长期糖尿病患者更易得肺结核。因此，在随访糖尿病患者时筛检其肺结核感染极为必要。

Translated from English version into Chinese by Zhang Zheng-yan, through

CHINESE CENTER FOR DISEASE CONTROL AND PREVENTION
NATIONAL INSTITUTE FOR PARASITIC DISEASES

Des cas de tuberculose pulmonaire à microscopie positive chez les patients diabétiques de l'hôpital central de Dessie, au nord-est de l'Ethiopie

Hiwot Amare, Aschalew Gelaw, Belay Anagaw, Baye Gelaw

Résumé

La tuberculose est une maladie infectieuse qui demeure la principale source de morbidité et mortalité à travers le monde. Les personnes atteintes de diabète sucré ont trois fois plus de risques de contracter une tuberculose évolutive que des personnes non-diabétiques. Cependant, il n'existe pas suffisamment d'informations fiables sur le fléau de la tuberculose pulmonaire chez les patients atteints de diabète sucré en Ethiopie, et principalement à Dessie. C'est pourquoi cette étude a pour objectif de déterminer la prévalence et les facteurs de risque associés à la tuberculose pulmonaire à microscopie positive chez les patients diabétiques de l'hôpital central de Dessie. Une étude transversale a été menée de février 2012 à avril 2012. Les caractéristiques démographiques des patients ont été rassemblées à l'aide d'un questionnaire type déjà testé par le passé. Des échantillons de crachats le matin à jeun ont été prélevés sur les patients participant à l'étude et examinés à la recherche de bacilles acido-résistants à l'aide d'une microscopie directe grâce à la technique de coloration de Zielh-Neelsen. Les données ont été saisies et analysées grâce au logiciel de statistiques SPSS version 16, et la valeur $p<0,05$ était considérée comme importante d'un point de vue statistique. Sur 225 patients diabétiques soupçonnés d'être atteints de tuberculose, 52 % étaient des hommes et 48 % des femmes. Leur âge variait entre 12 et 82 ans, avec un âge moyen de 47,2 ans. La prévalence de la tuberculose pulmonaire à microscopie positive était de 6,2 %. Résidence urbaine (Rapport de risque (RR) : 5.5 ; intervalle de confiance (IC) à 95% : 1.07 à 28.20), antécédents de tuberculose (RR : 13.4 ; IC à 95 % : 2.74 à 65.73), contact avec des patients atteints de tuberculose dans la famille (RR: 9.4 ; IC à 95 %: 1.82 à 48.50), et diabète sucré de longue durée (RR : 8.89 ; CI à 95 % : 1.88 à 58.12), tous ces facteurs ont été indépendamment associés au développement de la tuberculose évolutive chez les personnes vivant avec le diabète sucré. La prévalence de la tuberculose pulmonaire à microscopie positive était bien plus élevée chez les personnes diabétiques soupçonnées d'être atteintes de la tuberculose que dans l'ensemble de la population (respectivement 6,2 % et 0,394 %). Les patients ayant des antécédents de tuberculose, des contacts avec des personnes

atteintes de la tuberculose ou un diabète de longue durée étaient plus enclins à contracter une tuberculose pulmonaire. Par conséquent, lors du suivi des patients diabétiques, il est nécessaire de faire des tests de dépistage de la tuberculose pulmonaire infectieuse.

Translated from English version into French by chrystelle252, through

TRANSLATORS
WITHOUT BORDERS

Туберкулёз лёгких с позитивным мазком среди больных диабетом пациентов в Специализированной больнице г.Дэссе (Северо-Восточная Эфиопия)

Хивот Амаре, Ашалев Гелав, Белай Анагав, Байе Гелав

Краткое содержание

Туберкулёз – инфекционное заболевание, остающееся основной причиной заболеваемости и смертности во всём мире. Риск развития активного туберкулёза у людей с сахарным диабетом в три раза выше, чем у людей, не страдающих указанным заболеванием. Тем не менее, существует недостаток достоверной информации о распространённости туберкулёза лёгких среди больных сахарным диабетом как в Эфиопии в целом, так и в г.Дэссе в частности. Настоящее исследование направлено на выявление распространённости и факторов риска туберкулёза лёгких с позитивным мазком среди больных диабетом пациентов в Специализированной больнице г.Дэссе. Перекрёстное исследование проводилось с февраля по апрель 2012 года. Демографические характеристики пациентов собирались посредством апробированных стандартных вопросных форм. Тройные образцы мокроты собирались у участников исследования и изучались на наличие кислотоустойчивых бактерий посредством прямой микроскопии методом окраски по Цилю-Нельсону. Информация вводилась и анализировалась с использованием статистического программного обеспечения SPSS версии 16, p-значение <0.05 рассматривалось как статистически значимое. Из 225 подозревавшихся в заражённости туберкулёзом диабетиков 52% были мужчины и 48% - женщины. Их возраст варьировался от 12 до 82 лет, средний возраст составил 47,2 лет. Распространённость туберкулёза лёгких с позитивным мазком составила 6,2%. Проживание в городе (AOR: 5.5; 95%CI: 1.07-28.20), история туберкулёза (AOR: 13.4; 95%CI: 2.74-65.73), контакт с больными туберкулёзом в семье (AOR: 9.4; 95%CI: 1.82-48.50), а также длительность сахарного диабета (AOR: 8.89; 95%CI: 1.88-58.12) были независимо ассоциированы с развитием активного туберкулёза у людей, страдающих сахарным диабетом. Распространённость туберкулёза лёгких с позитивным мазком составила 6,2% у подозреваемых в заражённости туберкулёзом диабетиков, что выше по сравнению с населением в целом (0,394%). Пациенты, имевшие контакт с больными туберкулёзом и страдающие от диабета в течение

длительного времени, оказались более подвержены туберкулёзу лёгких. Исходя из вышеуказанного, при наблюдении больных диабетом пациентов необходимо их обследование на наличие туберкулёзной инфекции.

Translated from English version into Russian by OSU2011, through

TRANSLATORS
WITHOUT BORDERS

Frotis positivo de tuberculosis pulmonar en pacientes diabéticos del hospital de referencia en Dessie, en el noreste de Etiopia

Hiwot Amare, Aschalew Gelaw, Belay Anagaw, Baye Gelaw

Resumen

La tuberculosis es una enfermedad infecciosa que sigue siendo la principal causa de morbosidad y mortalidad en todo el mundo. El riesgo de desarrollar una tuberculosis activa es tres veces más alto en personas con diabetes mellitus que en aquellas personas sin diabetes. No obstante, en Etiopía en general y en Dessie en particular, no existe suficiente información verosímil sobre el problema de la tuberculosis pulmonar en pacientes con diabetes mellitus. Por eso, este estudio pretende determinar la prevalencia y los factores de riesgo asociados del frotis positivo de tuberculosis pulmonar en pacientes diabéticos del hospital de referencia en Dessie. Desde febrero hasta abril de 2012, se llevó a cabo un estudio transversal en el que se reunieron características demográficas de los pacientes, empleando para ello un formato de cuestionario estándar que ya había sido probado anteriormente. Se recogieron muestras matinales de esputo de los participantes del estudio y se estudiaron en busca de bacilos ácido-resistentes mediante microscopia directa, aplicando la técnica de tinción de Ziehl-Neelsen. Se utilizó la versión 16 del software para estadísticas SPSS para introducir y analizar los datos y el valor $P < 0,05$ se consideró una significación estadística. De los 225 pacientes diabéticos sospechosos de padecer TB, el 52 % eran hombres y el 48 % mujeres. Su edad estaba comprendida entre los 12 y los 82 años, siendo la media 47,2 años. La prevalencia del frotis positivo de tuberculosis pulmonar era de 6,2 %. Los residentes en zonas urbanas (AOR: 5,5; 95 % CI: 1.07-28,20), el historial de tuberculosis (AOR: 13,4; 95 % CI: 2,74-65,73), el contacto con pacientes de TB en la familia (AOR: 9,4; 95 % CI: 1,82-48,50) y la diabetes mellitus de larga duración (AOR: 8,89; 95 % CI: 1,88-58,12) estaban asociados de manera independiente con el desarrollo de la tuberculosis activa en personas afectadas de diabetes mellitus. La prevalencia del frotis positivo de tuberculosis pulmonar era de 6,2 % en pacientes diabéticos sospechosos de padecer TB, lo que representa un alto porcentaje si lo comparamos con el de la población general (0,394 %). Los pacientes con un historial previo de contacto con pacientes tuberculosos y aquellos con diabetes prolongada eran más propensos a padecer tuberculosis pulmonar.

Por lo tanto, es necesaria la selección de pacientes diabéticos para infecciones de tuberculosis pulmonar durante el seguimiento.

Translated from English version into Spanish by Nothomb, through

TRANSLATORS
WITHOUT BORDERS
